# Genetic Landscape of Open Chromatin in Yeast

**DOI:** 10.1371/journal.pgen.1003229

**Published:** 2013-02-07

**Authors:** Kibaick Lee, Sang Cheol Kim, Inkyung Jung, Kwoneel Kim, Jungmin Seo, Heun-Sik Lee, Gireesh K. Bogu, Dongsup Kim, Sanghyuk Lee, Byungwook Lee, Jung Kyoon Choi

**Affiliations:** 1 Department of Bio and Brain Engineering, Korea Advanced Institute of Science and Technology, Daejeon, Korea; 2 Korean Bioinformation Center, Korea Research Institute of Bioscience and Biotechnology, Daejeon, Korea; 3 Research Institute of Bioinformatics, Omicsis, Daejeon, Korea; 4 Center for Genome Science, National Institute of Health, Cheongwon, Korea; 5 Genome Institute of Singapore, Singapore, Singapore; Stanford University, United States of America

## Abstract

Chromatin regulation underlies a variety of DNA metabolism processes, including transcription, recombination, repair, and replication. To perform a quantitative genetic analysis of chromatin accessibility, we obtained open chromatin profiles across 96 genetically different yeast strains by FAIRE (formaldehyde-assisted isolation of regulatory elements) assay followed by sequencing. While 5∼10% of open chromatin region (OCRs) were significantly affected by variations in their underlying DNA sequences, subtelomeric areas as well as gene-rich and gene-poor regions displayed high levels of sequence-independent variation. We performed quantitative trait loci (QTL) mapping using the FAIRE signal for each OCR as a quantitative trait. While individual OCRs were associated with a handful of specific genetic markers, gene expression levels were associated with many regulatory loci. We found multi-target *trans*-loci responsible for a very large number of OCRs, which seemed to reflect the widespread influence of certain chromatin regulators. Such regulatory hotspots were enriched for known regulatory functions, such as recombinational DNA repair, telomere replication, and general transcription control. The OCRs associated with these multi-target *trans*-loci coincided with recombination hotspots, telomeres, and gene-rich regions according to the function of the associated regulators. Our findings provide a global quantitative picture of the genetic architecture of chromatin regulation.

## Author Summary

Quantitative trait loci (QTL) mapping is a genetic approach that allows the identification of genetic factors underlying a phenotype of interest. Genomic technologies such as DNA microarray and next-generation sequencing provide data that can be used for the analysis of multiple molecular phenotypes. For example, the expression levels of thousands of genes can be associated with subject-specific genome-wide genetic information in expression QTL mapping. Similarly, the genetic regulation of transcription factor binding or epigenetic mechanisms such as DNA methylation or chromatin structure has begun to be investigated. In particular, the mechanisms controlling chromatin accessibility have attracted special interest due to their importance in a variety of DNA regulation processes including recombination, repair, replication, and transcription. In this work, we sought to dissect the genetic architecture of chromatin accessibility regulation by harnessing the power of genetic and genomic techniques. By analyzing open (accessible) chromatin maps of multiple yeast individuals in association with their genetic backgrounds, we were able to characterize the regulatory structure of chromatin traits versus that of gene expression. Importantly, we observed that the genetic loci responsible for multiple open chromatin regions were enriched for known regulatory factors.

## Introduction

The genetic basis of gene expression has been studied in various organisms [1]–[5]. For example, two different strains of *Saccharomyces cerevisiae* (BY and RM) were crossed to produce a number of different genetic recombinants, and their expression levels and genotypes were analyzed [1], [6]. We previously utilized this system to separate the *cis*- and *trans*-components of variation in gene expression [7]. Tirosh *et al*. [8] profiled nucleosome patterns in the inter-specific hybrids of two yeast species to dissect *cis*- and *trans*-effects on nucleosome positioning. Recently, variations in the binding patterns of transcription factors (TFs) have begun to be studied [9]–[11].

Chromatin structure controls the access of a wide spectrum of DNA binding proteins involved in not only transcription but also DNA repair, recombination, and replication. Therefore, open chromatin areas can indicate DNA regions accessible to such regulators and thus have been used to identify regulatory regions or elements in the genome. In addition to the well-known DNaseI hypersensitivity assay, the FAIRE technique has been used to capture open chromatin sites in the genome with the aid of massively parallel sequencing (FAIRE-seq) [12]–[14]. In a recent study, the FAIRE DNA was analyzed by genotyping arrays to identify functional regulatory polymorphisms [15]. FAIRE-seq, however, is capable of providing a quantitative measure of chromatin accessibility along with sequence polymorphisms so that the direct effects of DNA sequences on chromatin accessibility can be examined. For example, it has been shown that SNPs located within open chromatin can influence chromatin accessibility, thus demonstrating that chromatin structure can be a heritable feature [11].

As chromatin is a genetically regulated material, a genetic association approach could be used to understand the genetic architecture of chromatin regulation by examining open chromatin in multiple genetically different individuals. A recent study [16] used this approach for chromatin accessibility across 70 human individuals. Because of the large size of the human genome, open chromatin sites were analyzed only in association with local genetic markers to identify *cis*-associations. Transcription factor binding was shown to be one of the main mechanisms by which DNA polymorphisms affect chromatin structure.

In this work, we took advantage of the compact size and comprehensive annotation of the yeast genome to dissect the entire genetic architecture of chromatin regulation, including both *cis*- and *trans*-associations, to better interpret the functional association of *trans*-acting factors. To this end, we generated open chromatin maps of 100 yeast samples, including the parental strains (BY and RM, two replicates of each) and their descendants [6] by means of the FAIRE-seq technique.

## Results

### General characterization of open chromatin regions

Open chromatin peaks were first identified for each sample. We then obtained a total of 7,527 OCRs by combining the peak signals of the 96 genetically different yeast strains. For each OCR, the density of the corresponding peak in each strain was calculated and normalized across the strains. The normalized peak density measures showed high reproducibility (R = 0.95∼0.99) between the replicates from different FAIRE batches and sequencing libraries (Figure S1). More than half of the OCRs were located at promoters, and 18.6% and 16.4% of the peaks fell near transcription termination sites and within ORFs, respectively (Figure S2). The OCRs mostly coincided with nucleosome-free regions at promoters or transcription termination sites (Figure S3). Approximately 57% of yeast genes contained an OCR at their promoter, and 40% of replication origins overlapped with 14.3% of the OCRs (Figure S2). The average size of the OCRs in BY and RM was 159 bp, while the average size of the OCRs combined across all the strains was 236 bp (Figure S4).

### Comparison of *cis*- and *trans*-variation

**Figure 1:**
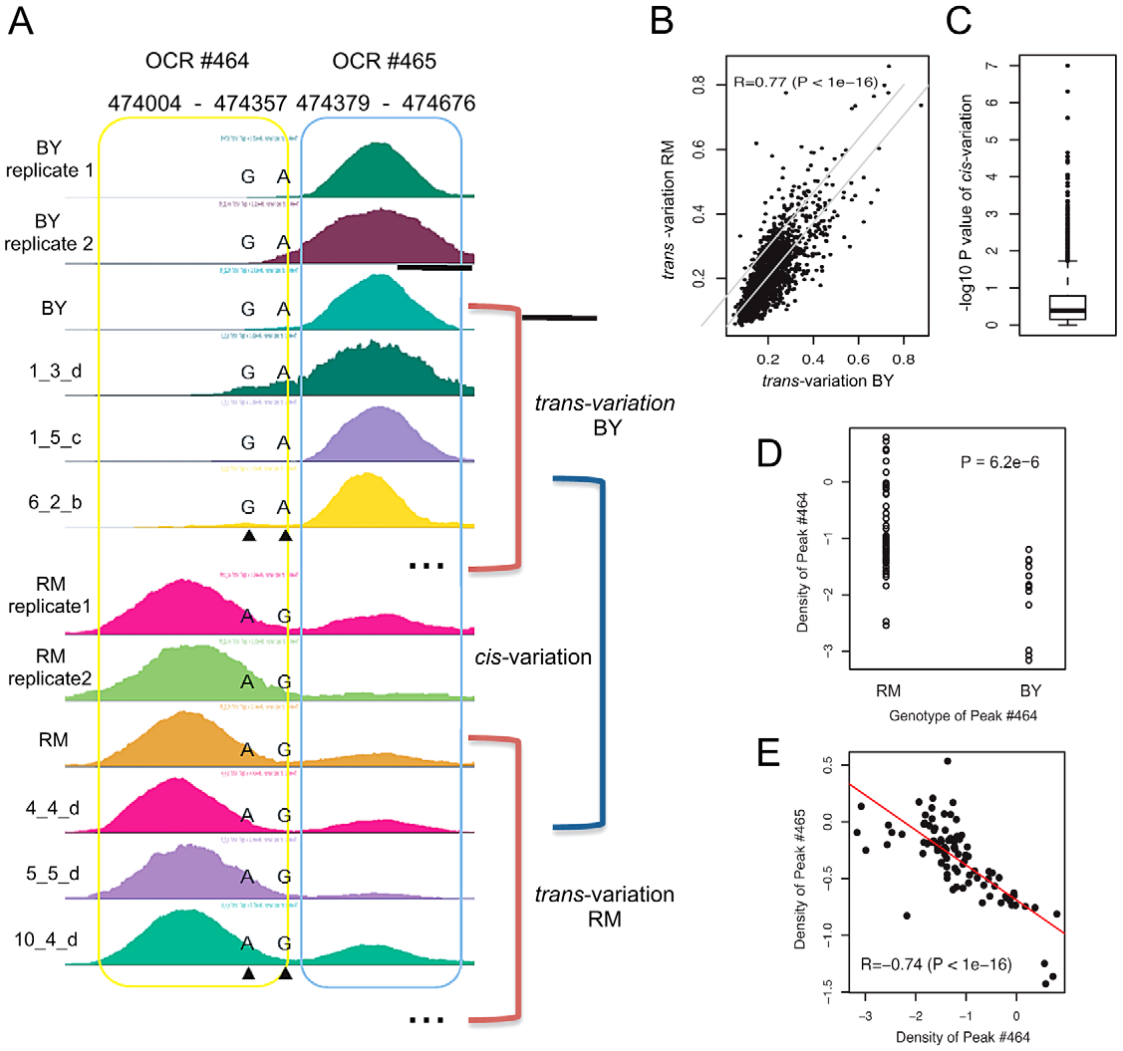
Measurement of *trans*- and *cis*-variation. (A) Sequence effects on chromatin regulation. The two peaks (OCR #464 and OCR #465) are shown for strains with the BY genotype and RM genotype, as determined based on the two SNPs found within OCR #464. (B) The two *trans*-variation measures were obtained as illustrated in Figure 1A and compared with each other. (C) The significance of *cis*-variation was measured by the t-test for the 1,738 OCRs. (D) Peak density of OCR #464 as a function of its genotype. (E) Anti-correlation between the peak density of OCR #464 and that of OCR #465 across all yeast strains.

We sought to estimate the direct influence of underlying DNA sequences on chromatin configuration by quantitatively comparing sequence-dependent (*cis*) variation and sequence-independent (*trans*) variation in chromatin accessibility. C*is*-variation indicates variation in chromatin accessibility among individuals in which the DNA sequences of the given open-chromatin locus are different, while *trans*-variation indicates variation in chromatin accessibility among individuals with an identical genotype at the given locus. To measure *cis*-variation as the magnitude of chromatin variation caused primarily by *cis*-acting elements residing directly beneath open chromatin, we sought to determine the genotype of each OCR based on the SNP profiles generated from our sequence data. This enabled the classification of OCRs into either BY or RM groups according to each strain's inheritance of the locus (Figure 1A). The *cis*-variation of each OCR was defined as the variance of peak density among the strains with the same genotype at that OCR. The two *cis*-variation measures (each from the BY and RM group) were highly consistent (Figure 1B). Approximately 23% (1,738 OCRs) had more than ten individuals in each group. We assessed the statistical significance of *trans*-variation by considering the within-group variance (*cis*-variation): 11.8% (P<0.05) or 4.8% (P<0.01) of the 1,738 OCRs were called significant (Figure 1C).

### *Cis*- and *trans*-associations in QTL mapping

QTL mapping was performed by interrogating the 7,527 OCRs against the genetic markers selected and processed based on the previous genotype data [6] (see Materials and Methods). A total of 11,048 associations were identified at a false discovery rate (FDR) of 0.01 by our chromatin QTL mapping. Approximately 7.9% of the associations involved *cis*-acting loci within 100 kb (12.66% within 1 Mb), whereas the majority of chromatin traits were linked to *trans*-regulatory loci. The OCRs associated in *trans* tended to display a higher *trans*-variation (P<2×10^−16^), while those associated in *cis* had a higher *cis*-variation (P = 1.1×10^−4^), indicating consistency between sequence-based genotyping and microarray-based genotyping. We employed the gene expression data for the 96 strains [6] and carried out expression QTL mapping by repeating the procedures used for the chromatin QTL mapping (see Materials and Methods). At an FDR of 0.01, 12,317 associations between genotypes and expression levels were identified.

### Characterization of *cis*-associations

We identified a total of 2,234 OCRs in which there was a TF-binding motif that contained a polymorphism and found that these OCRs were twice as likely to be associated in *cis* than other OCRs (P = 4.6×10^−7^). However, there was no difference with respect to *trans*-association. This implies that the effect of DNA sequence variation on chromatin structure is often manifested through underlying TF-binding motifs independently of *trans*-acting regulators.

To determine whether *cis*-associations can also be explained by differential nucleosome formation, we searched for *cis*-QTL SNPs in the well-known poly A/T tract nucleosome depletion signature. We extracted the reference genome sequences surrounding the SNP locations within the OCRs from our FAIRE-seq data and then looked for the presence of a poly A/T tract. Even with a very loose threshold (five consecutive A/Ts), we could only identify five such instances. This is contradictory to the major role of the AT-rich sequences in the divergence of nucleosome positioning between different species [8]. We propose that poly A/T tracts residing in open chromatin may be under strong selective pressure and thus resistant to sequence changes because of their importance in regulatory function.

Because the *cis*-associations between DNA sequences and chromatin accessibility are likely to be mediated by TF binding, a sequence polymorphism that affects chromatin accessibility in *cis* should also affect gene expression in the neighborhood. Indeed, a sizeable fraction (45%) of the chromatin-associated SNPs were associated with the expression of nearby genes. By contrast, only 15% of the expression-associated SNPs turned out to influence the accessibility of nearby chromatin, indicating that there are mechanisms by which sequence polymorphisms can affect the expression of nearby genes without affecting chromatin accessibility.

Reciprocal regulation of two chromatin loci by DNA sequences could be observed in OCR #464 and OCR #465. These two OCRs were associated with multiple *cis*-markers encompassing 100 kb upstream to 15 kb downstream of the loci. Sequence analysis detected two underlying SNPs that were associated with the peak density of OCR #464 (Figure 1A and 1D). Interestingly, the density of the adjacent peak (OCR #465) was negatively correlated with that of OCR #464 across the strains (Figure 1E), demonstrating a reciprocal regulation of the two chromatin loci. In line with our sequencing-based genotypes, all the *cis*-markers indicated that the RM genotype increases the peak density of OCR #464.

### Characterization of *trans*-associations

**Figure 2:**
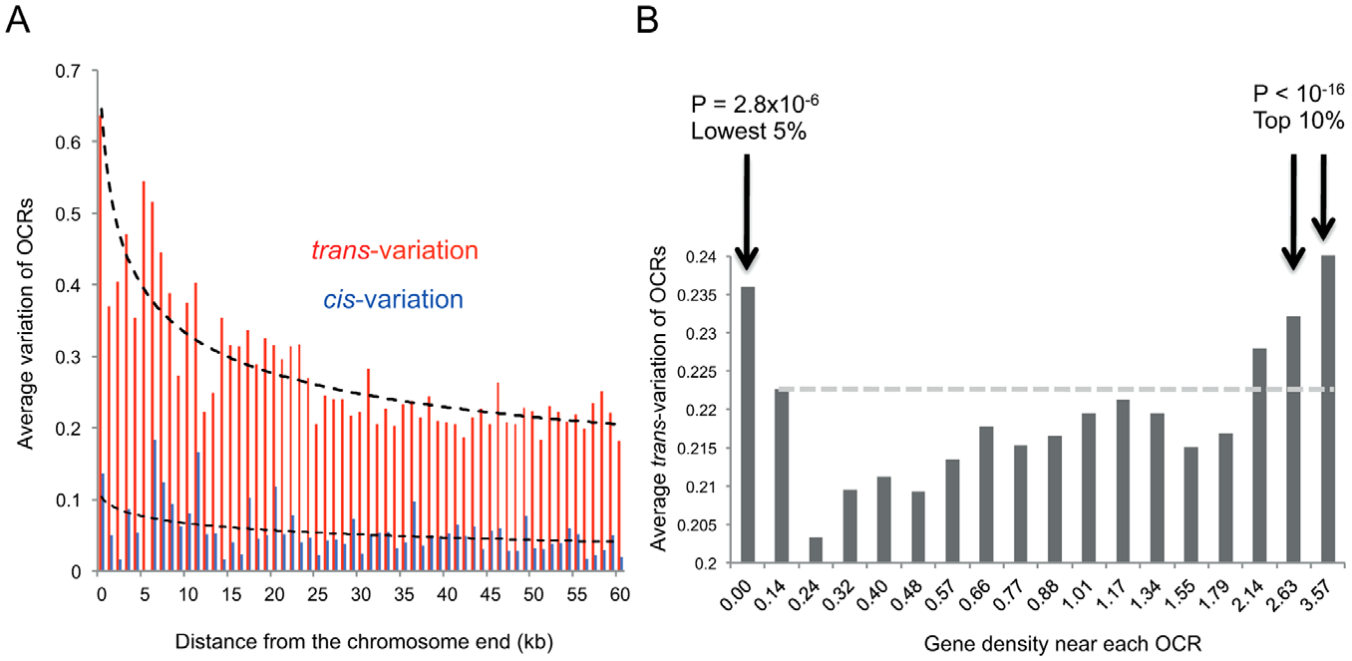
The magnitude of *trans-* and *cis-* variation and the number of genes within 50 kb upstream and downstream of the peak boundaries. (A) The magnitude of *trans*- and *cis*-variation as a function of the distance from chromosome ends. The average variation of OCRs within 2 kb windows was plotted for 1 kb bins. The *trans*-variations within 10 kb of the chromosome ends were significantly higher than those farther away (P<6.6×10^−25^). For *cis*-variation, the P value was 5×10^−4^ when the t-test was used. (B) The number of genes falling within 50 kb upstream and 50 kb downstream of the peak boundaries was obtained for each OCR. This number was divided by the size of the peak for the normalized gene density. Gene-rich OCRs (top 10%) and gene-poor OCRs (lowest 5%) were compared with the other OCRs by the t-test.

The sum of *trans*-variation in the *trans*-associated OCRs was divided by the sum of *trans*-variation across all the OCRs, revealing that 45.2% of the total *trans*-variation across the OCRs could be explained by genetic factors. To examine how much of the *trans*-variation of each OCR is explained by *trans*-acting genetic factors, we computed the explanatory power of the linear regression (R^2^) for each OCR and its associated *trans*-loci. The average R^2^ of the *trans*-associated OCRs was 33%. Enrichment of high *trans*-variation OCRs was observed in the vicinity of telomeres (Figure 2A and green marks in Figure S5). This pattern was not observed for *cis*-variation (Figure 2A and Figure S5). High *trans*-variation OCRs also coincided with gene-rich regions (Figure 2B and blue ticks in Figure S5) and gene-poor regions (Figure 2B and light-blue ticks in Figure S5).

Approximately 50% of chromatin QTLs were gene expression QTLs and vice versa, indicating that the *trans*-associations we identified are technically robust and biologically meaningful. However, only 17.6% of these dual QTLs were associated with chromatin and expression traits at the same locus. In other words, many of the dual QTLs were responsible for chromatin traits and gene expression traits that are distantly located (e.g., in different chromosomes). It is possible that regulatory SNPs affect chromatin accessibility for DNA regulation other than transcription (e.g., DNA repair, recombination, etc.), which in turn leads to secondary gene expression changes, and that regulatory loci affect the expression of downstream regulators in *trans*, which in turn causes secondary changes in the accessibility of the target chromatin regions.

**Figure 3:**
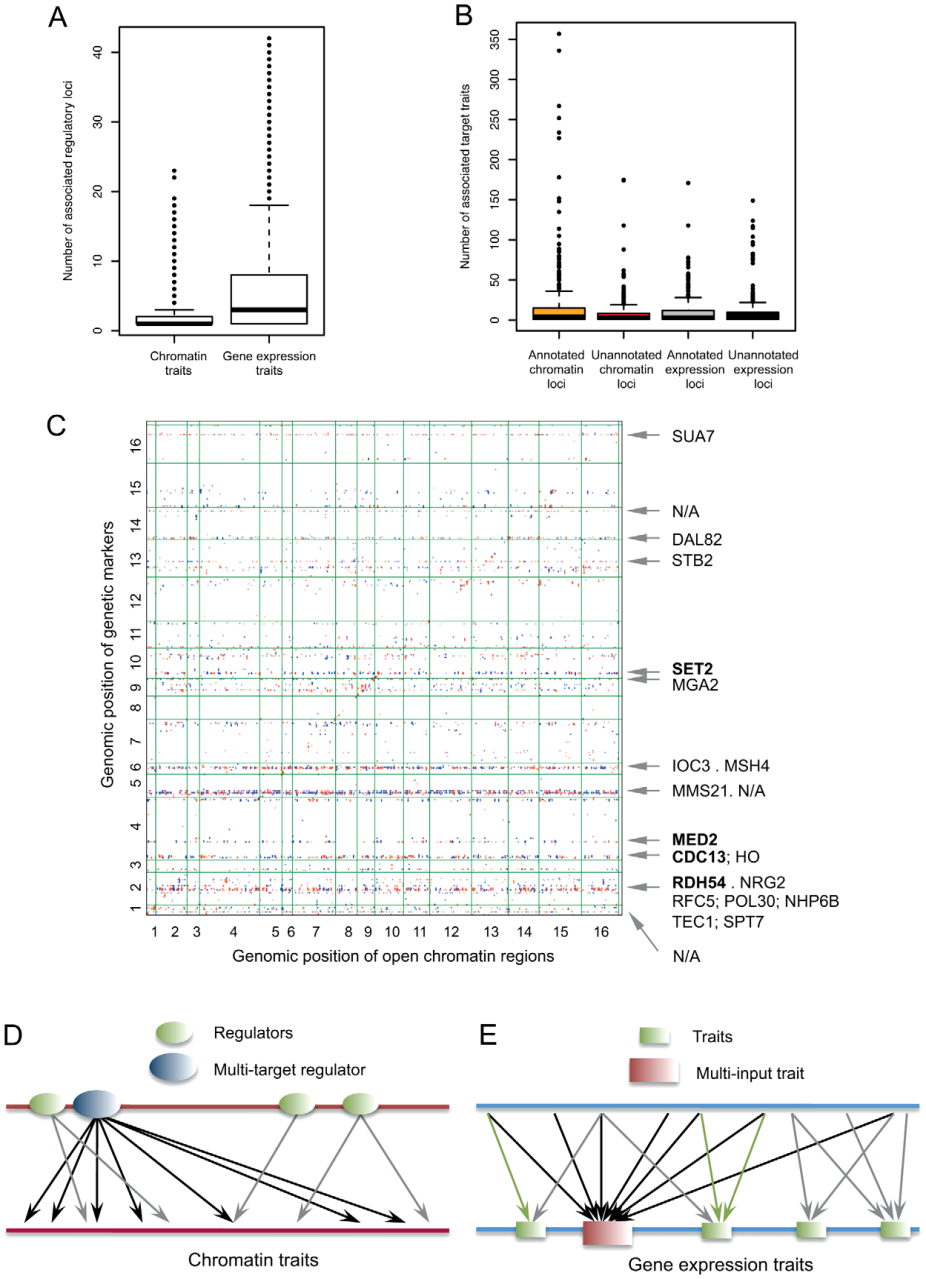
Characterization of *trans*-associations. (A) The number of *trans*-regulatory loci associated with each chromatin trait (left) and gene expression trait (right). (B) The number of target traits of each *trans*-regulatory locus was examined for chromatin QTLs and expression QTLs. Annotated QTLs were defined as having at least one known regulator in the vicinity. (C) In this chromatin association map, each dot indicates a linkage between a genetic marker (QTL; y axis) and a trait (OCR; x axis); red or blue indicates that the BY or RM genotype positively regulates the OCR, respectively. The annotation of the 17 QTL hotspots is shown on the right side. The names of the regulators associated with the same genetic marker are separated by a semicolon and those associated with closely located markers by a dot. N/A denotes an unannotated QTL. (D–E) Different regulation architectures of chromatin traits (D) and gene expression traits (E). On the regulator side, most chromatin regulatory loci are responsible for a few traits; however, certain regulatory loci can have upwards of 100 targets. On the target side, individual chromatin traits are usually targeted by less than five loci. The average number of associated loci is three times higher for gene expression traits than for chromatin traits, an indication that the transcription process is responsive to more regulatory inputs or stimuli.

We examined the number of *trans*-linkages for each OCR. Most OCRs were responsive to a small number of regulatory loci. Only a few (6.8%) had more than five linkages with the average number being three times lower than for gene expression traits (2.1 versus 5.9) (Figure 3A). This implies that chromatin traits are rather specifically governed by a handful of *trans*-regulators, whereas gene expression processes are responsive to more regulatory inputs. An opposite trend was observed for regulatory loci (Figure 3B). There were regulatory loci responsible for an extremely large number of chromatin traits, with a few cases in which >200 OCRs were linked to a single promiscuous chromatin QTL (Figure 3B). The horizontal dots observed in the chromatin association map (Figure 3C) illustrate ‘extensive’ regulation by chromatin regulatory loci (Figure 3D), as opposed to the ‘intensive’ regulation of gene expression traits (Figure 3E).

To investigate the multi-target chromatin regulatory loci, or hotspot QTLs, we first selected those with >65 *trans*-associated OCRs. We annotated each locus by searching for known DNA or chromatin regulators flanking the marker within 10 kb [17] and merged the adjacent markers covering the same regulator. A total of 32 initial hotspot loci were merged into 17 hotspots, 14 of which flanked at least one known regulator (master regulators listed in Figure 3C). The annotated (regulator-containing) loci tended to influence more chromatin traits than the unannotated loci (P = 5×10^−4^) (Figure 3B). By contrast, no enrichment of known regulators near multi-target expression regulatory loci was observed (Figure 3B).

**Figure 4:**
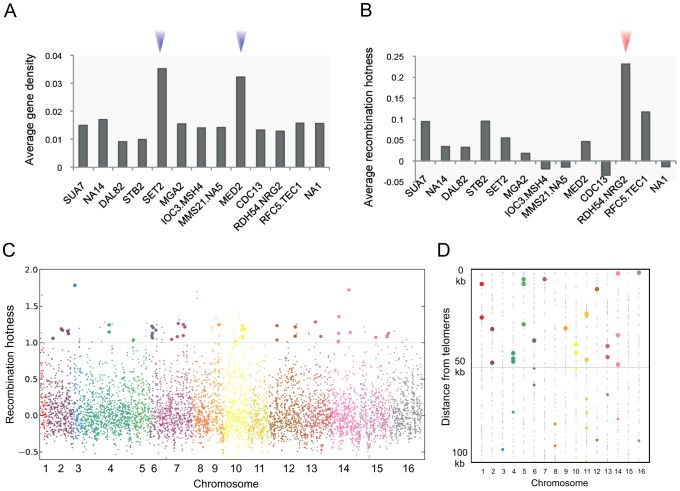
Functional analysis of the OCRs of multi-target regulators. (A–B) The average (A) gene density and (B) recombination hotness score (log2 ratio) for the OCRs associated with the multi-target regulators listed in Figure 3C. Unannotated QTLs were denoted as NA concatenated with the chromosome number (e.g., NA14 is on chromosome XIV). (C) Each spot corresponds to a genomic locus having a score for recombination hotness. Loci with a hotness score >1 located near the *RDH54*-associated OCRs are highlighted. (D) The dots indicate the OCRs of the multi-target regulators. The *CDC13* OCRs are colored according to the chromosome they belong to. The *CDC13* OCRs within 50 kb of telomeres are highlighted.

Among the master regulators (Figure 3C) were three TFs with sequence-specific DNA binding activity: *DAL82*, *TEC1*, and *NRG2*. Position weight matrices were available for the DNA-binding motif of Dal82p and Tec1p. Remarkably, 62% of the 71 *DAL82*-associated OCRs contained the Dal82p-binding motif. However, no Tec1p-binding motif enrichment was observed in the associated OCRs. The influence of Tec1p might be exerted not through direct binding but via interaction with other factors under normal growth conditions. Data for Nrg2p binding sites are not available. *SET2* and *MED2* are involved in the transcription of many genes in a non-sequence-specific manner. Set2p is a histone methyltransferase that plays a role in general transcription elongation, and Med2p is a subunit of the mediator complex that forms the RNA polymerase II holoenzyme. Their target OCRs were identified in gene-rich regions (Figure 4A and Figure S6).

Rdh54p is a Swi2/Snf2-like factor that plays a role in recombinational repair of DNA double-strand breaks (DSBs) during mitosis and meiosis by interacting with Rad51p and Rad54p [18]–[20]. DSBs occurring at recombination hotspots in yeast are found near open chromatin [21]. We employed a measure of “recombination hotness” that was globally obtained based on DSB distribution [22]. The *RDH54* OCRs showed the highest recombination hotness among the master regulators (Figure 4B), with a P value of 9×10^−25^ (Figure S7), and tended to fall near the recombination hotspots (Figure 4C). Cdc13p is a multi-functional telomere-binding protein that participates in telomere replication and maintenance especially by mediating telomerase access to telomeric chromatin [23]–[25]. Among the hotspot loci, the *CDC13* locus had the largest number of associated OCRs in close proximity to telomeres (seven OCRs within 1 kb from telomeres). The enrichment of *CDC13*-associated OCRs near telomeres is shown in Figure 4D. Telomeres are associated with recombination coldspots [22]. Indeed, the recombination hotness of the *CDC13* OCRs was very low (Figure 4B).

## Discussion

In this work, we sought to dissect the genetic architecture of chromatin regulation. The multi-target regulatory structure reflects the wide-ranging nature of certain chromatin regulators. Surprisingly, however, many chromatin QTLs were found to govern only a few target traits. It is conceivable that the chromatin structures at particular loci are not susceptible to genetic perturbations or that the technical limitations of our method for detecting subtle changes in chromatin traits may prevent the identification of weakly associated targets. In this case, there may be numerous potential regulatory targets that have not passed our statistical threshold.

On the other hand, the chromatin traits that were responsive to certain genetic perturbations had only a few regulatory inputs, in contrast to the high responsiveness of gene expression traits to multiple regulatory signals. Therefore, chromatin states alone may not be sufficient to explain the precise level of transcription. Once upstream regulators set the stage by priming the chromatin structure, various downstream regulatory inputs may add additional layers of complexity to gene expression control. This is also reflected in the lack of common targets between chromatin QTLs and expression QTLs. Only 18% of the dual QTLs (i.e., SNPs that are both chromatin QTLs and expression QTLs) were associated with chromatin accessibility and gene expression at the same locus simultaneously. However, the identification of many dual QTLs was encouraging itself because it suggests that the detected QTLs are likely to contain functional regulators. We successfully annotated chromatin QTLs, particularly those responsible for a large number of target chromatin traits. The identification of functionally relevant *trans*-regulators from expression QTL mapping has been reported to be difficult [26].

Sequence-specific TF binding appears to be very important in *cis*-associations. We observed an enrichment of *cis*-associations for TF-motif-containing OCRs and common QTLs linking chromatin accessibility and nearby gene expression. This is consistent with the finding that human SNPs associated with chromatin in *cis* are frequently found in TF-binding sites [16]. Moreover, consistency in allele frequencies were observed between the sequence reads for open chromatin and those for TF binding.

In contrast to the previous study [16] in which only *cis*-regulation was thoroughly examined, here we took advantage of the compact size and comprehensive annotation of the yeast genome to dissect the architecture of *trans*-regulatory mechanisms as well. In conclusion, our work provides insight into the genetic basis of chromatin regulation and its relationship with transcription control. Genetic variation in open chromatin in the human genome can underlie disease phenotypes, and thus, the current study has medical implications. For example, previous studies [13], [15] identified regulatory polymorphisms in open chromatin that were previously linked through genome-wide association studies with diabetes and HDL cholesterol levels.

## Materials and Methods

### Identification of OCRs and estimation of peak density

We obtained the BY-RM cross strains from the original authors [1], [6]. FAIRE experiments were performed based on the published protocol [12]. We selected 94 yeast segregants and subjected them and the BY and RM strains to 100-bp sequencing on Illumina HiSeq2000. To identify the FAIRE-seq read peaks, we ran F-Seq [27] as previously suggested for FAIRE-seq data analysis [13]. Small-sized peaks (<15 bp) were extended in both directions such that all the peaks were at least15 bp long. To identify all possible OCRs, we combined the extended peaks of the 96 yeast strains (exclusive of the replicates) and merged overlapping peaks into a single peak using BEDTools [28], resulting in 7,527 unique OCRs. The number of FAIRE-seq reads that mapped uniquely to each OCR was counted in each yeast strain. The read count of each OCR was normalized by taking into account the size of the peak and the total number of tags produced from each FAIRE library as 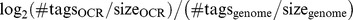. After the log2 transformation, the negative values were set to zero (ceiling). This normalization scheme was used in our previous work [29]. We further normalized the final matrix of the 7,527 OCRs and 96 strains by scaling the 96 sample vectors to zero mean and unit variance. To assess reproducibility, the FAIRE-seq reads of the parental replicates were mapped to the predefined OCRs and the same normalization scheme was repeated for the four independent samples.

### Genotyping of the OCRs and estimation of *trans-* and *cis*-variation

SNPs were detected from the FAIRE-seq reads using the Illumina's CASAVA suite. SNP calls with fewer than five reads were discarded. For heterogeneous calls, only the major polymorphism with a certain frequency (>80%) was taken. The genotype of each OCR was determined based on its SNP profile. The OCR in the given strain was considered to have inherited the BY (or RM) allele if its genotype perfectly matched with the genotype of the OCR in the BY (or RM) strain. For genotyping at a less stringent threshold, the OCRs whose SNP profile matched with either the BY or RM profile for >50% of the SNPs were also classified as BY or RM. To compute *trans*-variation, the standard deviations of the normalized peak density measures within the BY and RM groups was measured. We identified a total of 1,738 OCRs for which at least ten individuals inherited either a BY or RM allele; we then re-grouped the yeast strains according to the genotype of the given OCR. To assess the statistical significance of *cis*-variation, we used the two-sample t test to measure the difference in the means of the BY and RM groups.

### Chromatin QTL mapping and expression QTL mapping

The genetic markers from the original study [6] were used for QTL mapping. As suggested by Lee et al. [17], adjacent markers with no more than two genotypic mismatches across the 96 strains were merged into one average genotype profile, resulting in 1,533 markers. As suggested previously [17], we identified the genes located within 10 kb upstream or downstream of the genomic region covered by the merged genetic marker. To identify potential regulators, we used Gene Ontology to identify 495 genes involved in “DNA binding”, and 508 genes known to be involved in transcription and chromatin regulation, resulting in a total of 752 unique genes. For QTL mapping, we measured associations by means of the correlation coefficient or the linear regression between the genotypes represented as a categorical variable (0: RM, 0.5: missing, 1: BY) and the chromatin traits represented as the normalized peak-density measure. False discovery rates (FDRs) were computed based on the permutation test, as follows. The matrix of peak density was shuffled by resampling the sample vectors (yeast strains) to generate 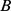 randomized matrices, 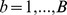
_._ The P value was determined by comparing the observed association 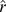 with the expected associations 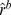 from the permuted data as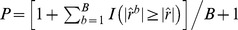, where 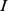 is an interpretation function. 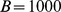 was used. The P values were adjusted for multiple testing to yield FDRs, as suggested by Benjamini and Hochberg [30]. An FDR of 0.01 was used. A distance of 100 kb between the marker and the trait was used to differentiate *cis*- and *trans*-associations. We employed the gene expression data for the 96 strains [6] and performed expression QTL mapping by repeating the same procedures.

### Analysis of chromatin QTL mapping results

A total of 11,048 marker-trait associations involving 3,522 OCRs were identified at an FDR of 0.01 when the correlation coefficient was used. To evaluate the consistency between the FDR-based non-parametric approach and the parametric method, we obtained a P value for each marker-trait pair based on the linear regression. At parametric P values<10^−3^ and <10^−5^, 91.1% and 81.1% of the identified associations were called significant, respectively. Adjacent genetic markers (<10 kb) associated with a common trait in QTL mapping were combined. *Trans*-loci were examined to determine whether the corresponding genetic marker covered at least one of the 752 regulators. According to this criterion, all *trans*-loci were classified into annotated loci or unannotated loci. We defined hotspot chromatin loci as having more than 65 genetic linkages. Adjacent genetic markers covering the same regulator were manually merged.

### Additional data analysis

To calculate the density of the genes surrounding each OCR, the number of genes located within 50 kb upstream and 50 kb downstream of the OCR peak boundaries was determined. This number was divided by the size of the peak for normalization. The microarray data for the recombination hotspots of the yeast genome [22] were downloaded from http://derisilab.ucsf.edu/hotspots/. The cy5/cy3 ratios from seven ORF arrays were averaged and log2 transformed. The positions of replication origins were downloaded from http://cerevisiae.oridb.org. For TF motif analysis, we used position weight matrices [31] based on *in vivo* binding assays by chromatin immunoprecipitation for 203 yeast TFs [32] and another set of position weight matrices based on systematic *in vitro* assays of 112 yeast TFs [33]. TF motifs occurring in OCRs were identified by means of the HOMER package [34] using the two position weight matrix sets.

### Data availability

The FAIRE-seq data for the 96 yeast strains are available at the GEO database with accession number GSE33466.

The following link has been created to allow review of the record GSE33466: http://www.ncbi.nlm.nih.gov/geo/query/acc.cgi?token=zvyznqwickewmto&acc=GSE33466

## Supporting Information

Figure S1 Reproducibility of FAIRE-seq. Beside BY and RM FAIRE-seq run on HiSeq2000, an additional set of FAIRE-seq libraries was independently sequenced on Illumina GA2. Another set of FAIRE samples was separately prepared and sent to another sequencing operator for library preparation and sequencing on Illumina GA2. In total, we sequenced three different batches of FAIRE-seq libraries for each of BY and RM. The normalized peak density of the OCRs from HiSeq2000 sequencing was compared with the two replicates from the completely different batches.
(PDF)

Figure S2 The percentage of OCRs falling on the promoter, ORF, and transcription termination site of protein-coding genes and on replication origins.
(PDF)

Figure S3 The frequency of OCRs (gray shade) found near the transcription start site (left panel) and the transcription termination site (right panel) in comparison with nucleosome occupancy (black curve).
(PDF)

Figure S4 The size of the OCRs identified in either parental strain (BY or RM) and those combined across the 96 strains (BY, RM, and their 94 descendants).
(PDF)

Figure S5 Chromosome-wide maps of *trans*-variation. The magnitude of *trans*-variation for each OCR was plotted along with the chromosomal coordinates of telomeres, centromeres, gene-rich or gene-poor regions, *CDC13*-associated OCRs, and multi-input OCRs (those with more than five associated QTLs).
(PDF)

Figure S6 Gene density surrounding the OCRs of the master regulators as listed in Figure 3C. The number of genes within 50 kb upstream and 50 kb downstream of the peak boundaries of each OCR was obtained and divided by the size of the peak. Unannotated loci were denoted as NA concatenated with the chromosome number (e.g., NA14 is on chromosome XIV).
(PDF)

Figure S7 Recombination hotness of the OCRs of the master regulators as listed in Figure 3C. Shown is –log10 of the P value of the one-sample t statistic to test if the hotness scores are less than zero. Unannotated loci were denoted as NA concatenated with the chromosome number (e.g., NA14 is on chromosome XIV).
(PDF)
