# Supplementary figures and images for: Genetic Landscape of Open Chromatin in Yeast
Source: PLoS Genet. 2013 Feb 7;9(2):e1003229. doi: 10.1371/journal.pgen.1003229 (PMC3567132; doi:10.1371/journal.pgen.1003229)

Figure S1

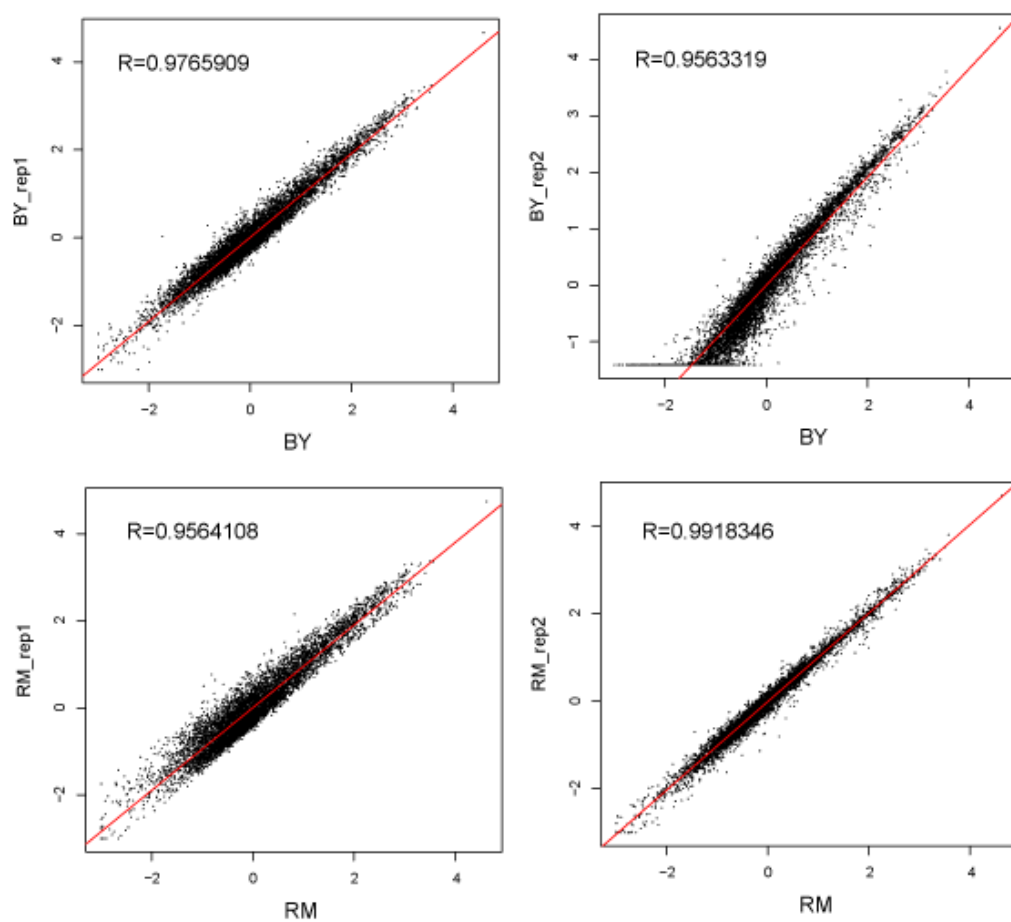

Supplement: Figure S1 — Reproducibility of FAIRE-seq. Beside BY and RM FAIRE-seq run on HiSeq2000, an additional set of FAIRE-seq libraries was independently sequenced on Illumina GA2. Another set of FAIRE samples was separately prepared and sent to another sequencing operator for library preparation and sequencing on Illumina GA2. In total, we sequenced three different batches of FAIRE-seq libraries for each of BY and RM. The normalized peak density of the OCRs from HiSeq2000 sequencing was compared with the two replicates from the completely different batches. (PDF) [file pgen.1003229.s001.pdf]

Figure S2

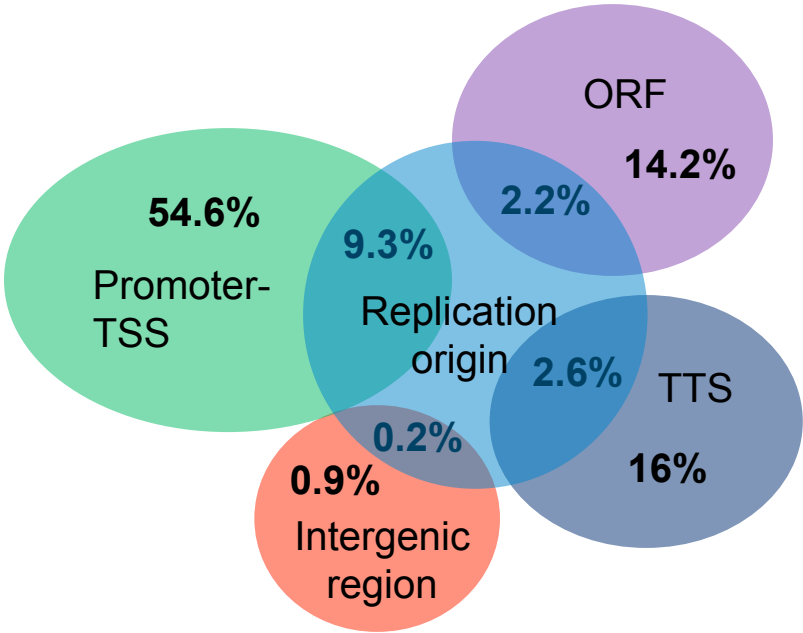

Supplement: Figure S2 — The percentage of OCRs falling on the promoter, ORF, and transcription termination site of protein-coding genes and on replication origins. (PDF) [file pgen.1003229.s002.pdf]

Figure S3

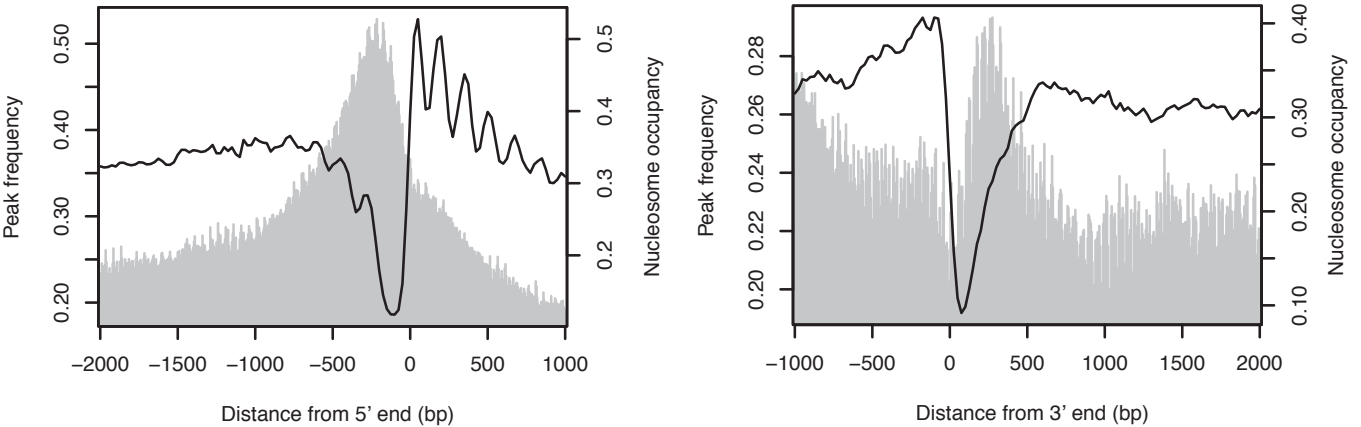

Supplement: Figure S3 — The frequency of OCRs (gray shade) found near the transcription start site (left panel) and the transcription termination site (right panel) in comparison with nucleosome occupancy (black curve). (PDF) [file pgen.1003229.s003.pdf]

Figure S4

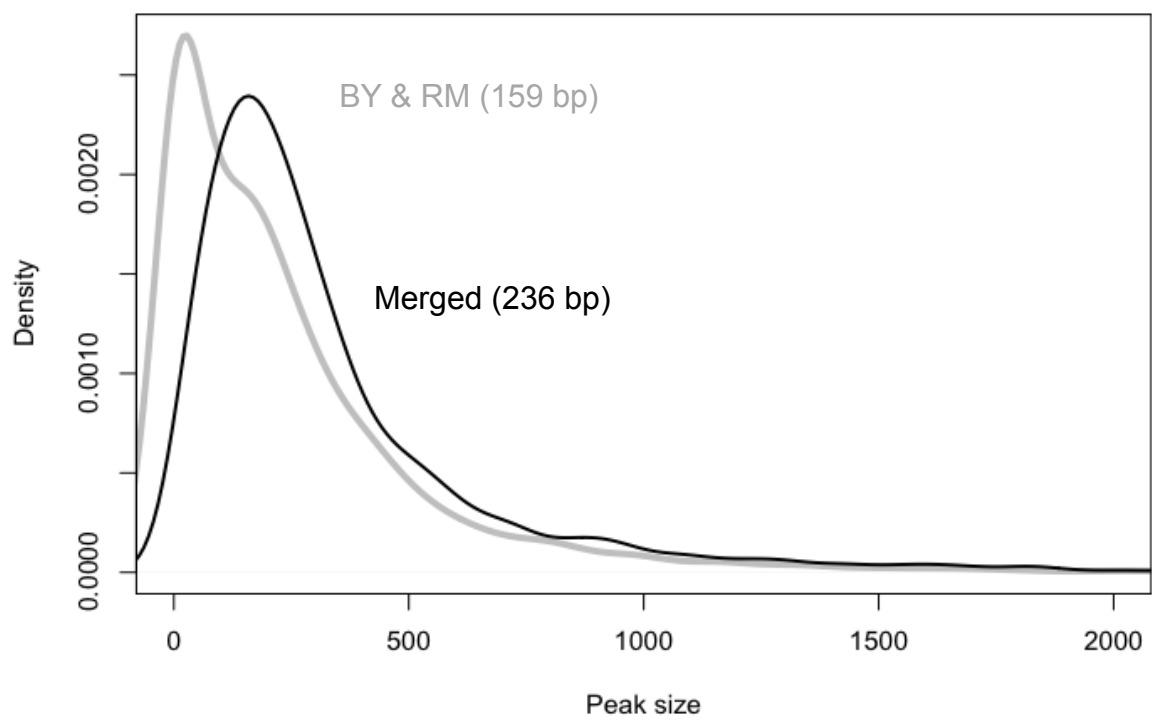

Supplement: Figure S4 — The size of the OCRs identified in either parental strain (BY or RM) and those combined across the 96 strains (BY, RM, and their 94 descendants). (PDF) [file pgen.1003229.s004.pdf]

Figure S5

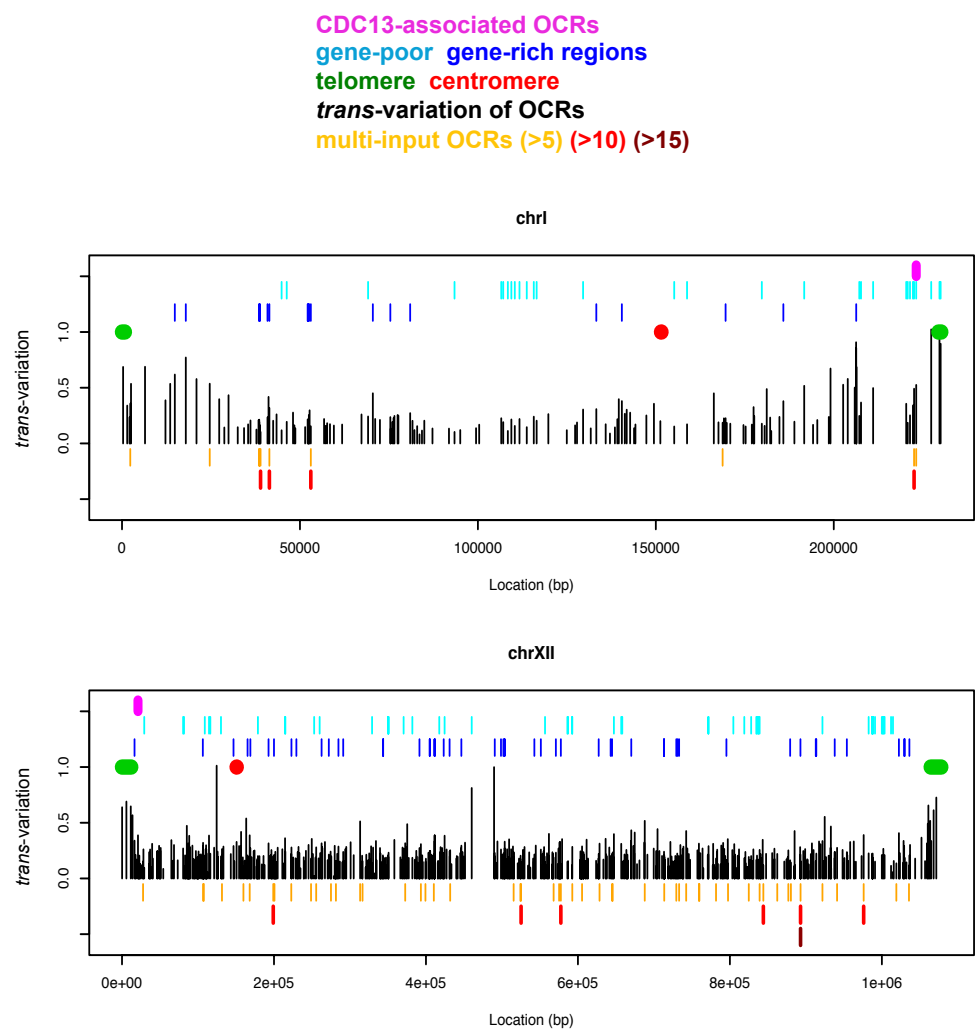

Supplement: Figure S5 — Chromosome-wide maps of trans-variation. The magnitude of trans-variation for each OCR was plotted along with the chromosomal coordinates of telomeres, centromeres, gene-rich or gene-poor regions, CDC13-associated OCRs, and multi-input OCRs (those with more than five associated QTLs). (PDF) [file pgen.1003229.s005.pdf]

Figure S6

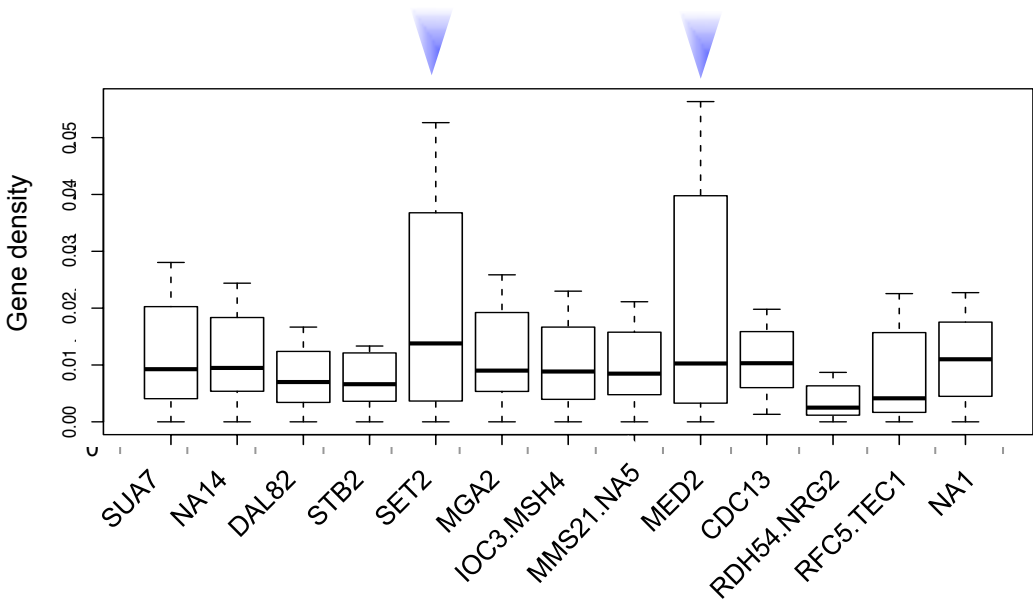

Supplement: Figure S6 — Gene density surrounding the OCRs of the master regulators as listed in Figure 3C. The number of genes within 50 kb upstream and 50 kb downstream of the peak boundaries of each OCR was obtained and divided by the size of the peak. Unannotated loci were denoted as NA concatenated with the chromosome number (e.g., NA14 is on chromosome XIV). (PDF) [file pgen.1003229.s006.pdf]

Figure S7

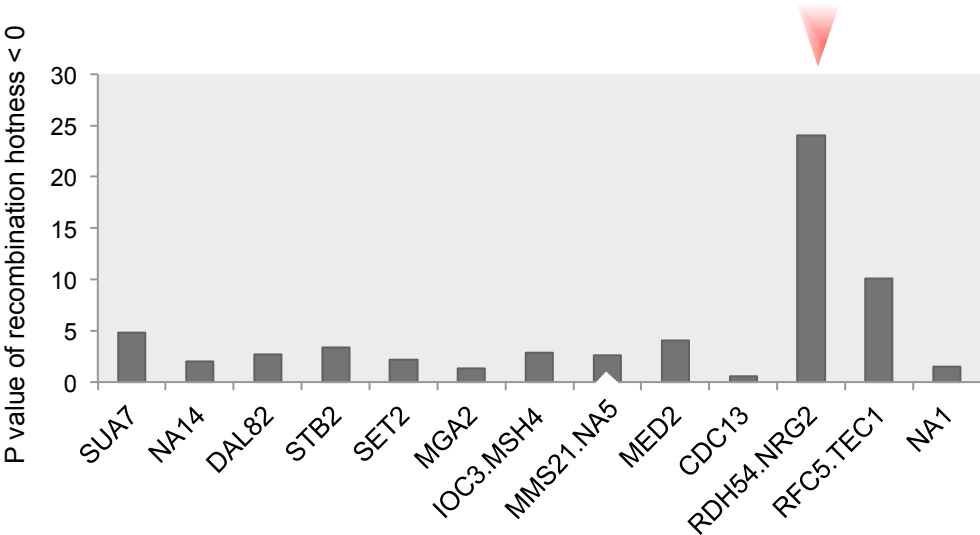

Supplement: Figure S7 — Recombination hotness of the OCRs of the master regulators as listed in Figure 3C. Shown is –log10 of the P value of the one-sample t statistic to test if the hotness scores are less than zero. Unannotated loci were denoted as NA concatenated with the chromosome number (e.g., NA14 is on chromosome XIV). (PDF) [file pgen.1003229.s007.pdf]
